# Time-restricted eating for prevention of age-related vascular cognitive decline in older adults: A protocol for a single-arm open-label interventional trial

**DOI:** 10.1371/journal.pone.0314871

**Published:** 2024-12-09

**Authors:** Ana Clara da C. Pinaffi-Langley, Zsofia Szarvas, Anna Peterfi, Zalan Kaposzta, Peter Mukli, Ali Shahriari, Mihaly Muranyi, Camila B. Pinto, Cameron D. Owens, Cheryl Adams, Brittany Karfonta, Michael Rohan, Stefano Tarantini, Andriy Yabluchanskiy

**Affiliations:** 1 Oklahoma Center for Geroscience and Healthy Brain Aging, University of Oklahoma Health Sciences, Oklahoma City, OK, United States of America; 2 Department of Nutritional Sciences, College of Allied Health, University of Oklahoma Health Sciences, Oklahoma City, OK, United States of America; 3 Vascular Cognitive Impairment and Neurodegeneration Program, Department of Neurosurgery, University of Oklahoma Health Sciences, Oklahoma City, OK, United States of America; 4 Oklahoma Shared Clinical and Translational Resources, University of Oklahoma Health Sciences, Oklahoma City, OK, United States of America; 5 Laureate Institute for Brain Research, Tulsa, OK, United States of America; 6 Department of Health Promotion Sciences, College of Public Health, University of Oklahoma Health Sciences, Oklahoma City, OK, United States of America; 7 Peggy and Charles Stephenson Cancer Center, University of Oklahoma Health Sciences, Oklahoma City, OK, United States of America; Public Library of Science, UNITED KINGDOM OF GREAT BRITAIN AND NORTHERN IRELAND

## Abstract

Age-related cerebromicrovascular endothelial dysfunction underlies the initiation and progression of cognitive dysfunction and dementia, thus increasing the susceptibility of older adults to such conditions. Normal brain function requires dynamic adjustment of cerebral blood flow to meet the energetic demands of active neurons, which is achieved the homeostatic mechanism neurovascular coupling (NVC). In this context, therapeutical strategies aimed at rescuing or preserving NVC responses can delay the incidence or mitigate the severity of age-related cognitive dysfunction, and time-restricted eating (TRE) is a potential candidate for such a strategy. Studies have reported that TRE can improve cardiometabolic risk factors in older adults. However, the effect of TRE on cerebrovascular endothelial function remains unexplored. Thus, this protocol outlines the study procedures to test our hypothesis that a 6-month TRE regimen of 10-h eating window will improve NVC responses and endothelial function in community-dwelling older adults. This is a single-arm, open-label interventional trial. We aim to recruit 32 adults aged 55–80 years. Participants are instructed to maintain a TRE regimen of 10 h of free eating followed by 14 h of fasting for 6 months. Before and after fasting, participants are assessed for cognitive performance, peripheral micro- and macrovascular endothelial function, and NVC responses, as well as for several confounding factors, including body composition, dietary, and physical activity data. We expect that 6 months of TRE will improve NVC response and endothelial function in older adults compared with baseline, and that these improvements will be accompanied by improvements in cognitive performance. The study proposed herein will provide critical insight into a new potential therapeutical strategy for targeting age-related cognitive dysfunction. Ultimately, slowing down or alleviating cognitive decline will translate into improved quality of life and longer healthspan for aging adults. This study was prospectively registered at ClinicalTrials.gov (NCT06019195) on August 24, 2023.

## Introduction

Recent reports estimate 10% of US adults aged 65 years and older have dementia [1, 2], which corresponds to about 7 million people. This proportion is expected to increase as the population becomes older [3], representing a significant public health challenge. Age-related endothelial dysfunction contributes to the development and progression of cognitive impairment and dementia [4–6], thus increasing the susceptibility of older adults to such diseases. In particular, cerebromicrovascular endothelial dysfunction is associated with impaired cerebral blood flow, which in turn is associated with cognitive decline [7–10]. Normal brain function requires moment-to-moment adjustment of cerebral blood flow to meet the energetic demands of active neurons [11–13]. This requirement is achieved by neurovascular coupling (NVC), a homeostatic mechanism that contributes to an optimal cerebral tissue microenvironment. In this context, therapeutical strategies aimed at rescuing age-related cerebromicrovascular endothelial function can delay the incidence or slow the progression of cognitive impairment in older adults.

Dietary modifications are accessible non-pharmacological therapeutical strategies commonly prescribed in clinical settings. Among these, time-restricted eating (TRE) has garnered special attention due to its increased feasibility and acceptability compared with other common dietary interventions such as continuous caloric restriction. TRE regimens divide the day into eating (4–10 h) and fasting (20–14 h) periods; during eating periods, one can consume their total daily energy needs without restriction. To date, few studies on TRE in older adults (>50 years of age) have been published [14–21]. These studies consistently reported decreases in body weight and visceral fat mass, and improvements in glucose homeostasis. They also report good safety and tolerability for this type of intervention in older adults. However, studies published thus far were short, lasting between 3 to 12 weeks. Further, most studies have not conducted nutritional assessments during the intervention, which is a major confounding factor in TRE studies. In this way, the effect of long-term TRE on cerebrovascular function and cognitive performance in older adults remains unexplored. Herein, we describe the protocol of a study on TRE in older adults according to SPIRIT reporting guidelines [22] (S1 File).

## Aims and objectives

The main aim of this study is to determine the effect of a 6-month TRE regimen on cerebrovascular function, cognitive performance, and peripheral endothelial function in community-dwelling older adults. Thus, the study objectives are the following:

- Determine whether adhering to 6 months of TRE improves neurovascular coupling responses in older adults
- Determine whether adhering to 6 months of TRE improves micro- and macrovascular endothelial function in older adults
- Determine whether adhering to 6 months of TRE improves crystallized and fluid cognitive performance in older adults

## Materials and methods

### Trial design and status

This study is a single-arm, open-label interventional trial. This study opened for enrollment on on April 9, 2024. We expect to reach target enrollment by February 2025.

### Study setting

This is a single center study primarily set in the Translational Geroscience Laboratory at the University of Oklahoma Health Sciences (Oklahoma City, OK). Collaborating sites are the Oklahoma Clinical and Translational Science Institute (Oklahoma City, OK) and the Laureate Institute for Brain Research (Tulsa, OK). All recruitment and enrollment occur at the Translational Geroscience Laboratory. Collaborating sites perform additional assessments only.

### Participants

#### Ethical considerations

This study protocol was reviewed and approved by the University of Oklahoma Health Sciences Institutional Review Board (#16102) on July 12, 2023.

#### Eligibility criteria

Participants are considered eligible if they meet all the inclusion criteria and do not meet any of the exclusion criteria. The inclusion criteria are as follows: (1) age between 55 and 80 years, inclusive; (2) adequate hearing and visual acuity to participate in the assessments; (3) ability to read and write in English; (4) competence to provide written informed consent; (5) Mini Montreal Cognitive Assessment score equal or greater than 12; and (6) Mini Nutritional Assessment score equal or greater than 12.

The exclusion criteria are the following: (1) active central nervous system disease (e.g., multiple sclerosis, uncontrolled seizures, active brain cancer); (2) cerebrovascular accident other than transient ischemic attack in the preceding 2 months; (3) major uncontrolled or unmedicated psychiatric disease; (4) current alcohol or drug abuse; (5) diabetes diagnosis with prescription of sulfonylureas, meglitinides, and/or insulin; (6) any other condition that, in the opinion of the investigator, would render the participant ineligible for the study.

#### Sample size estimation

Our primary endpoint is change in neurovascular coupling response in older adults comparing baseline and endpoint. We estimated the effect size for sample size calculation using preliminary data acquired with our pilot study on TRE (10 h free eating, 14 h fasting) in adults aged 55 years and older (N = 6). Regression coefficients (β values) were estimated for channels in the prefrontal and dorsolateral cortexes (see Statistical methods section for details). The mean difference between endpoint and baseline was 7.4 with a standard deviation of 13.1, representing an effect size of 0.56. At 80% power and 5% significance level, we calculated that we would need 27 participants to detect a similar effect size. Considering a dropout rate of 20%, the final sample size is 32 participants. We will conduct both intention-to-treat and per-protocol analyses.

#### Recruitment and enrollment

**Fig 1:**
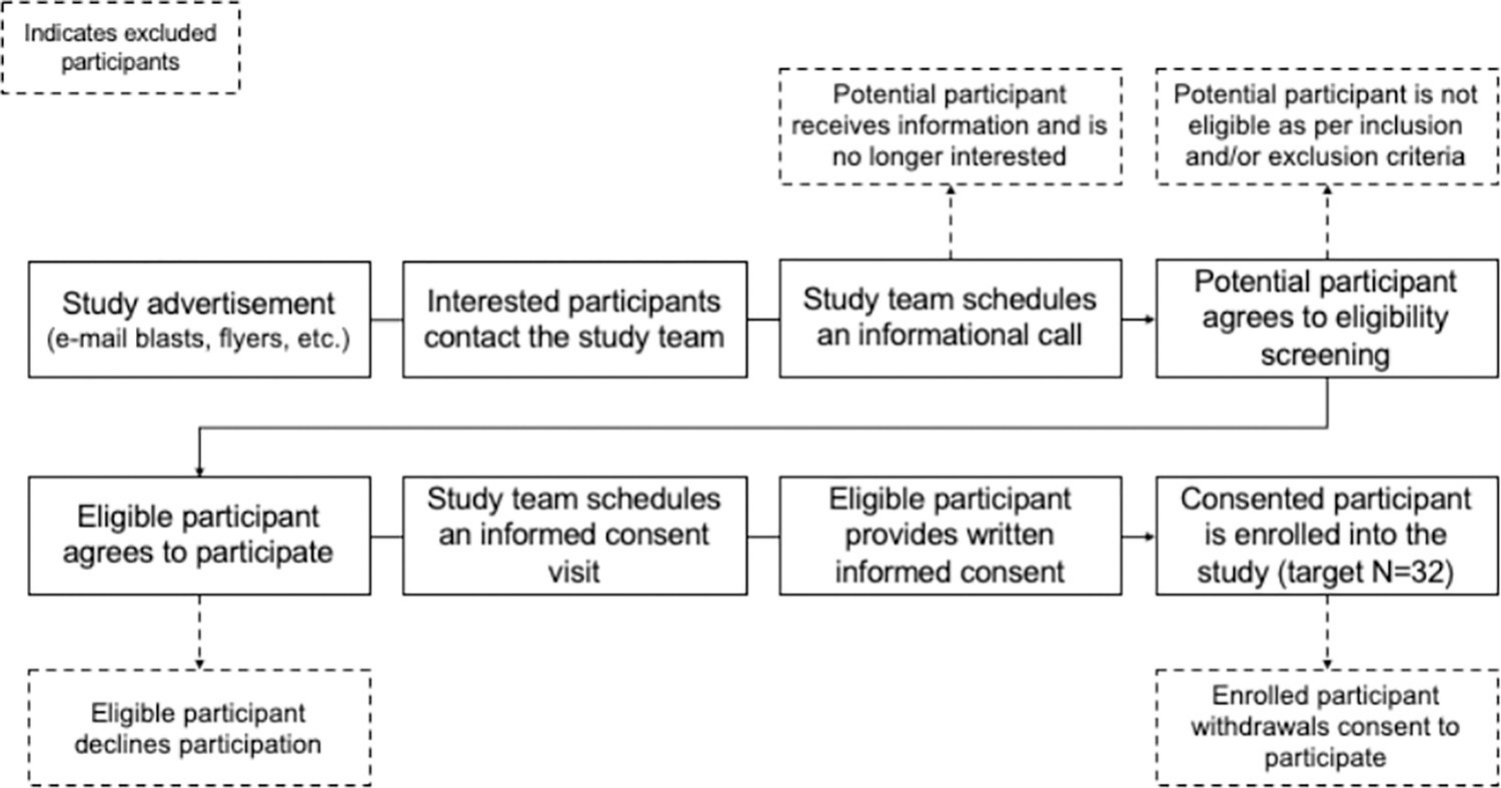
Participant recruitment and enrollment flowchart.

Participants are recruited using advertisements and partnerships. We use different types of advertisement to reach potential participants, such as electronic/virtual (e-mail blasts, social media), traditional media (local newspapers and magazines), and print media (flyers). Recruitment is also performed using an internal participant pool and partnerships with the Oklahoma Clinical and Translational Sciences Institute and the Section of Geriatric Medicine. This diverse recruitment strategy was devised to ensure an adequate number of participants for reaching the target sample size. Based on past enrollment rates, we estimate that we will need to screen at least 107 potential participants to achieve the target sample size. Eligible participants are considered enrolled in the study after they provide written informed consent. Only trained staff active in the IRB protocol obtain written informed consent. Consenting occurs in person and is conducted following institutional and ethical regulations. Fig 1 details the recruitment and enrollment process, including the scenarios for exclusion and/or screen fail.

### Intervention

The intervention is a time-restricted eating regimen that divides the day into 10 h of free eating and 14 h of fasting. Participants are instructed to maintain their usual dietary habits during the free eating period and to avoid skipping meals. Instead, they are instructed to compress their eating schedule into the 10 h of free eating to minimize concurrent caloric restriction. During the fasting period, participants are instructed to only consume water, unsweetened tea, or unsweetened coffee. The fasting period is self-selected so participants can choose what best fits their schedule and lifestyle. We believe that self-selection of the fasting period will increase retention and adherence, and that it represents a more generalizable approach to intermittent fasting studies. The intervention will last 26±2 weeks.

#### Adherence and monitoring

At the end of the first study visit, participants receive a fasting diary and are instructed to record the times of their first and last meal each day starting the following day. They are instructed to record these times even if they ate for more than 10 h or fasted for less than 14 h in any given day, and to return the complete diaries on their final study visit. These diaries will be used to estimate overall adherence to the time-restricted eating regimen.

Based on our previous experience with the pilot study and the literature on long-term dietary interventions [23], we expect that study retention will be a major challenge. To promote retention and minimize drop out or loss to follow up, we implemented a follow-up schedule for contacting active study participants every 2 weeks. During follow-up calls, participants are asked about any changes in health status and occurrence of adverse events, their fasting experience, and whether they have any questions or concerns regarding their study participation. If the participant expresses that the frequency of the contact is too high, it will be scaled back to once a month.

### Study outcomes

All outcomes are be assessed by trained study staff and research technicians.

#### Primary outcomes

The primary outcome of interest for this study is change from baseline in NVC response measured with functional near-infrared spectroscopy (fNIRS) during cognitive stimulation. Although the clinical meaning of NVC response is undetermined, NVC responses are positively correlated with cognitive performance in humans [24]. Thus, we expect that adhering to the intervention will increase NVC responses compared with baseline.

#### Secondary and exploratory outcomes

Secondary and exploratory outcomes for this study are described in Table 1. These outcomes include measures of micro- and macrovascular endothelial function, cognitive performance, functional connectivity, systemic inflammation and oxidative stress, and body composition.

**Table 1:** Summary of outcomes assessed during study visit 1 and study visit 2.

Outcome measure	Definition	Methodology
**Neurovascular coupling response** *	(1) Change in oxygenated and deoxygenated hemoglobin concentration in cortical tissue upon cognitive stimulation*	(1) Functional near-infrared spectroscopy
	(2) Change in retinal vessel diameter from baseline upon flickering light–induced dilation	(2) Dynamic vessel analyzer
**Band-limited power**	Change in electrocortical activity in distinct frequency bands upon cognitive stimulation	Electroencephalography
**Flow-mediated dilation in artery diameter**	Maximal change in brachial artery diameter from baseline upon reactive hyperemia	Ultrasonography
**Reactive hyperemia-induced fold change**	Maximal change in microvascular perfusion on the dorsal surface of the hand from baseline upon reactive hyperemia	Laser speckle contrast imaging
**Reactive hyperemia-induced acute perfusion**	Rate of change in microvascular perfusion on the dorsal surface of the hand during the first 4 sec of reactive hyperemia	Laser speckle contrast imaging
**Radial augmentation index**	Proportion of the central pulse pressure that results from the primary reflected wave and indicates arterial stiffness	Pulse wave analysis
**Glycocalyx-perfused boundary region**	Degree of red blood cell penetration into the glycocalyx; inversely correlated with glycocalyx thickness	Sublingual vascular assessment (GlycoCheck)
**Capillary density**	Total number of 10-micron long microvessel segments that occupy the recorded area	Sublingual vascular assessment (GlycoCheck)
**Red blood cell filling percentage**	Median percentage of time in which red blood cells are present in a microvessel (defined in the system at each measurement point)	Sublingual vascular assessment (GlycoCheck)
**BOLD activation** **	Change in concentrations of paramagnetic deoxygenated hemoglobin in specific regions of the brain upon cognitive stimulation	Functional magnetic resonance imaging**
**Pro-inflammatory cytokine levels**	Fluorescence intensity quantification of circulating cytokines in serum samples	Magnetic bead-based multiplex assay
**Blood lipid levels**	Quantification of blood lipids in whole blood samples using chemistry reagent discs (Lipid Panel Plus)	Blood chemistry analyzer (Piccolo Xpress, Abaxis)
**Fluid cognition**	Composite score including the scores of Dimensional Change Card Sort, Flanker Inhibitory Control and Attention, Picture Sequence Memory (Form A), List Sorting Working Memory, and Pattern Comparison tests	Cognitive battery (NIH Toolbox)
**Cognitive performance**	Fully adjusted scale scores of Picture Vocabulary, Flanker Inhibitory Control and Attention, List Sorting Working Memory, Dimensional Change Card Sort, Pattern Comparison Processing Speed, and Picture Sequence Memory tests. Raw score of Oral Symbol Digit test.	Cognitive battery (NIH Toolbox)
**Working memory performance**	Reaction time (ms) and accuracy index during the N-back paradigm	N-back paradigm
**Total fat mass**	Percentage of body weight corresponding to fat mass	Bioimpedance scan and/or dual X-ray absorptiometry*
**Total fat-free mass**	Percentage of body weight corresponding to the sum of body cell mass and extracellular mass (i.e., extracellular water)	Bioimpedance scan and/or dual X-ray absorptiometry*

* Indicates primary outcome

** Indicateas optional assessment

#### Confounding variables

In addition to the outcomes described above, we will also collect data on several confounding variables that may affect the measured outcomes. These confounding variables will be used in statistical analyses to normalize data and adjust models, as appropriate (see Statistical methods section for further details). Table 2 presents a summary of confounding variables along with their method and time of assessment.

**Table 2:** Summary of confounding variables assessed during the study period.

Confounding variable	Assessment method	Assessment time
**Age**	Demographic questionnaire	Visit 1
**Sex**	Demographic questionnaire	Visit 1
**Level of education**	Demographic questionnaire	Visit 1
**Race and ethnicity**	Demographic questionnaire	Visit 1
**Polypharmacy**	Medical history questionnaire, defined as the regular use of 5 or more medications (prescription or OTC)	Visit 1 and Visit 2 (update)
**Blood pressure**	Automatic sphygmomanometer	Visit 1 and Visit 2 (4 individual recordings per visit)
**Cardiovascular risk factors**	Medical history questionnaire	Visit 1 and Visit 2 (update)
**BMI**	Calculated using reported height and measured weight in a digital scale	Visit 1 and Visit 2
**Comorbidities**	Medical history questionnaire	Visit 1 and Visit 2 (update)
**COVID-19 infection history**	Medical history questionnaire	Visit 1 and Visit 2 (update)
**Physical activity level**	CHAMPS questionnaire	Visit 1 and Visit 2
**Estimated TDEE**	Calculated based on age, sex, measured fat mass, measured weight, and physical activity level	Visit 1 and Visit 2
**Average energy intake**	5-pass 24-h dietary recall	Twice a month until Visit 2 (total of 12 individual recalls)
**Weight**	Digital scale	Visit 1 and Visit 2
**Adherence to intervention**	Calculated using self-reported daily fasting timings (fasting diary) and defined as percentage of days with reported eating window no longer than 10 h and reported fasting window no shorter than 14 h	Daily until Visit 2
**Smoking status**	Medical history questionnaire	Visit 1 and Visit 2 (update)
**Alcohol use**	Medical history questionnaire	Visit 1 and Visit 2 (update)
**Emotional status**	NIH Toolbox (unadjusted scale scores of Perceived Stress, Self-Efficacy, General Life Satisfaction, and Loneliness tests)	Visit 1 and Visit 2

OTC: over the counter, BMI: body mass index, CHAMPS: Community Healthy Activities Model Program for Seniors, TDEE: total daily energy expenditure

### Data collection methods

Assessments described in the following subsections are repeated in Visits 1 and 2 unless otherwise stated.

#### Demographic, medical, and lifestyle-related assessments

During Visit 1, demographic and medical history information is collected during an interview with a study team member. Interviews are conducted in a private, dedicated room, and answers are entered directly into a secure data repository system (REDCap, Vanderbilt University). Demographic information includes sex, age, race, ethnicity, and education level. Medical history includes cardiovascular, endocrine, gastrointestinal, genitourinary, infectious, musculoskeletal, neurological, oncological, psychological, and respiratory conditions, as well as a complete list of current medications (both prescription and over the counter) and supplements. At this time, social history information is also collected (smoking status, alcohol use, and recreational drug use). During Visit 2, medical and social history information is updated as needed.

Information on physical activity is collected using the CHAMPS Physical Activity Questionnaire for Older Adults. This questionnaire is self-administered using a printed version, and it assesses the weekly frequency and duration in which one is engaged in a variety of physical activities [25]. The main measure derived from this assessment is estimated weekly caloric expenditure. Dietary intake data will be collected and analyzed using the Automated Self-Administered 24-hour (ASA24) Dietary Assessment Tool, version 2024, developed by the National Cancer Institute (https://epi.grants.cancer.gov/asa24). The ASA24 system does not capture any personally identifiable data from respondents and respondents’ data are protected by industry standard security protocols. The ASA24 tool is validated for use in the general US population and has comparable performance to interviewer-administered 24-h recalls [26, 27]. Participants are instructed to complete one 24-h recall biweekly for a total of 12 recalls. They are also instructed to perform the recalls on different days to cover different week and weekend days. If participants express that they cannot use the automated system, a trained study team member conducts interviewer-led dietary recalls over the phone instead. Recall data will be used to estimate participants usual dietary intake during the intervention.

#### Anthropometric assessments

Anthropometric data is collected using bioelectrical impedance analysis. Participants are instructed to empty their pockets and step onto the bioimpedance scale (BC-418, Tanita, Tokyo, Japan) while barefoot. After the weight measure stabilizes, participants hold the grips to the side of their bodies and remain still until the measurement is complete. All participants undergo bioelectrical impedance analysis while fasting at the beginning of the study visit, and they are given a chance to urinate prior to stepping onto the scale. As an optional procedure, participants may elect to undergo a whole-body dual X-ray absorptiometry (DEXA; Lunar iDXA with enCORE software version 17, GE Healthcare, Chicago, IL) [28]. This assessment occurs at the Oklahoma Clinical and Translational Science Institute (Oklahoma City, OK) up to two weeks after Visit 1 and up to two weeks before or after Visit 2.

#### Cognitive performance assessments

Cognitive performance data are collected using the NIH Toolbox Cognitive Battery (Toolbox Assessments, Inc., Chicago, IL) and the N-back working memory paradigm. The NIH Toolbox Cognitive Battery includes the following tests: Picture Vocabulary, Flanker Inhibitory Control and Attention, List Sorting Working Memory, Dimensional Change Card Sort, Pattern Comparison Processing Speed, Picture Sequence Memory, and Oral Symbol Digit. These tests cover several cognitive domains, including executive function, attention, memory, language, and processing speed. The NIH Toolbox Battery is administered using the iPad version in a quiet, dedicated room and takes about 45 minutes to complete. This tablet version is adequate for older adults (>54 years old) and repeated measures with an interval of at least 4 weeks [29].

The N-back task is administered during cerebrovascular assessments to elicit neurovascular responses (see next section for details on the cerebrovascular assessments) using E-Prime 3.0 software (Psychology Software Tools, Pittsburgh, PA). The N-back task has increasing difficulty levels and is related to working memory function. Briefly, participants watch an instructional video that explains how to perform the task, and the examiner verbally confirms that they understood the task and provides clarification as needed. For the task, the participant monitors several stimuli on a computer screen and engages with the computer program by clicking the left mouse button whenever they recognize the stimulus as equal to another one presented N steps previously, where N is a prespecified number. In the 1-back task, the participant should react when they recognize a letter that is the same as the letter presented 1 step back. For instance, if the participant is presented with the sequence of letters A-B-C-**C**-D, they should respond to the letter **C** sequence. In the 2-back task, the participant should react when they recognize a letter that is the same as the letter presented 2 steps back. For example, if the participant is presented with the sequence of letters A-B-C-**B**-D, they should respond to the letter **B** sequence. We conduct 0-, 1-, and 2-back tasks, where 0-back tasks are defined as the baseline condition (identification of the prespecified letter **W**). For cognitive stimulation during fNIRS and electroencephalography (EEG), each task contains 32 letters and lasts 72 seconds, and the sequence of tasks is 0-back, 1-back, 0-back, and 2-back, with 10 seconds of rest between each task, for a total of 4 blocks. For cognitive stimulation during fMRI, each task contains 20 letters and lasts 35 seconds. The sequence of tasks is the same but with 15 seconds of rest between each task and repeated 4 times for a total of 16 blocks. The cognitive performance parameters evaluated with the N-back task are reaction time (defined as the time in ms between stimulus presentation and response) and accuracy index (defined as the subtraction of the z-transformed scores for correct hit and false alarm rates) for each N-back task, as described elsewhere [24].

#### Physiological assessments

Single-point blood pressure is measured at pre-specified time points: before NIH Toolbox battery, before dynamic retinal vessel analysis, before laser speckle contrast imaging, and before brachial artery sonography. Blood pressure is also measured continuously during fNIRS and EEG. Single-point blood pressure measurements are taken with an automatic sphygmomanometer (upper arm blood pressure monitor) while participants are seated in an upright position according to clinical guidelines [30]. Continuous blood pressure measurements are taken with an automatic finger cuff system (Human NIBP Nano, ADInstruments, Sydney, Australia) and recorded on LabChart (ADInstruments). Arterial augmentation index is measured using radial artery tonometry and pulse wave analysis (SphygmoCorCVMS, AtCor Medical, New South Wales, Australia).

Flow-mediated dilation of the brachial artery was measured according to published guidelines [31]. Leads are placed on the participant’s torso for a 3-lead electrocardiography to monitor their heart rate continuously during the flow-mediated dilation procedure, and they are allowed to rest supine for at least 15 minutes prior to the measurement. A sphygmomanometer cuff is placed below the antecubital fossa on the right forearm in preparation for the occlusion phase. This cuff is attached to an automatic inflator (Hokanson E20 Rapid Cuff Inflator, Bellevue, WA) set to sustain pressure at 50 mmHg above the participant’s current systolic blood pressure or at a maximum of 200 mmHg. The right brachial artery is visualized using a Doppler ultrasound system equipped with an 8–12 MHz transducer (Phillips Affinity 70, Cambridge, MA). After acquiring 60 seconds of stable baseline imaging, reactive hyperemia is induced by sustaining forearm occlusion for 5 minutes and releasing it. Brachial artery diameter imaging is recorded for the last 30 seconds of occlusion and 2.5 minutes following cuff deflation. Imaging data is analyzed using Brachial Analyzer for Research software (Medical Imaging Applications LLC, Coralville, IA) to obtain artery diameter values and calculate the flow-mediated dilation response [31, 32].

Microvascular perfusion parameters (fold change and acute perfusion) are assessed using laser speckle contrast imaging (PSI System, Perimed, Jarfalla, Sweden) under reactive hyperemia conditions. Participants are seated upright with their left hand placed directly underneath the camera system. A sphygmomanometer cuff is placed proximal to the antecubital fossa on the left upper arm. Before and after the assessment, the temperature along the middle finger (nailbed, medial phalange, and metacarpo-phalangeal joint) is measured using a non-contact thermometer (TW2, Thermoworks, American Fork, UT). After acquiring 60 seconds of stable baseline, reactive hyperemia is induced in the same way as described for the brachial arterial flow-mediated dilation procedure. Continuous perfusion data is analyzed using PIMSoft software (Perimed) to calculate reactive hyperemia-induced fold change and acute perfusion [32].

Sublingual microvascular parameters (glycocalyx-perfused boundary region, capillary density, and red blood cell filling percentage) are obtained using a side-stream dark field camera (CapiScope HVCS, KK Technology, Honiton, UK) and GlycoCheck software (Microvascular Health Solutions Inc., Salt Lake City, UT). Participants are seated upright and instructed to swallow any excess saliva. The camera is then inserted into the participant’s mouth and held in place in the sublingual region for imaging recording. The software conducts quality control in real time, only recording for analysis images within quality parameters of focus, motion, and intensity. The recording takes 5 to 7 minutes to complete, and the analysis is automatically performed by the software.

Retinal vessel reactivity is assessed using a Dynamic Vessel Analyzer (IMEDOS, Jena, Germany) according to published protocols [33, 34]. The pupil of one eye is dilated using topical tropicamide (1% tropicamide ophthalmic solution USP) 20 minutes prior to the assessment. Using a mydriatic retinal camera, retinal arterial and venous diameters are continuously recorded during baseline and flickering light stimulation (total duration: 350 s). After recording 50 seconds of stable baseline, three cycles of stimulation are performed consecutively, each consisting of 20 seconds of flickering light (12.5 Hz) followed by 80 seconds of steady light (resting period). Imaging data is analyzed using the integrated software (IMEDOS) to obtain arterial vessel diameter values and calculate the reactivity parameters, as previously described [35, 36].

Neurovascular coupling responses and brain electrical activity are assessed simultaneously using fNIRS (NIRSport2, NIRx Medical Technologies, LLC, Glen Head, NY) and EEG during cognitive stimulation (N-back task, as described in the Cognitive performance assessments subsection). EEG data are recorded using a 16 channel biosignal amplifier (g.HIamp, g.tec medical engineering GmbH, Castleton-On-Hudson, NY) and 16 active electrodes placed according to the international 10–5 system on a tight fabric cap (EASYCAP GmbH, Herrsching am Ammersee, Germany). fNIRS data are acquired using 16 pairs of sources and detectors placed according to the international 10–20 system on the same fabric cap to form 48 channels and 8 short-distance channels. Sampling frequency for EEG and fNIRS data acquisition are 1200 Hz and 5.1 Hz, respectively. Temporal synchronization is achieved by simultaneous triggers using a parallel port.

As an optional procedure, participants may elect to undergo a fMRI scanning. Both structural (T1-weighted, T2, and tissue characterization scans) and functional (single-shot EPI sequence scan) imaging data are acquired in a 3.0 T magnetic resonance system. Functional runs include a resting state period (10 minutes) and a cognitive task (N-back task, as described in the Cognitive performance assessments subsection). This assessment occurs at the Laureate Institute for Brain Research (Tulsa, OK) up to two weeks after Visit 1 and up to two weeks before or after Visit 2.

#### Biospecimen collection

Up to 40 mL of blood is collected by trained staff into heparin, EDTA, and untreated tubes. Heparinized blood is used immediately upon collection for quantifying blood lipids using chemistry reagent discs (Lipid Panel Plus, Piccolo Xpress, Abaxis, Union City, CA). EDTA-treated and untreated blood samples are processed to separate plasma, serum, and buffy coat aliquots, which are stored in -80°C freezers (along with EDTA-treated whole blood aliquots) for further analysis.

#### Safety considerations

The procedures in this study incur no more than minimal risks that are associated with routine medical procedures. Nonetheless, some procedures have potential physical risks that require additional safety considerations. For dynamic vessel retinal analysis, physical risks include photosensitive epilepsy induced by the flickering light and acute glaucoma attack induced by pupil dilation. Therefore, participants undergo safety screening prior to the procedure, which includes a questionnaire to assess eye health (prior eye surgery, existing eye pathologies, tropicamide allergy, etc.) and seizure history, and eye examination to assess intraocular pressure (eligibility cutoff <22 mmHg; iCARE CI100 tonometer, Icare, Vantaa, Finland) and anterior chamber depth (slit lamp examination). If a participant has significant eye pathology, tropicamide allergy, positive seizure history, intraocular pressure above the cutoff value, and/or shallow anterior chamber, they are not eligible for dynamic vessel analysis and are excluded from the procedure. Similarly, participants who opt-in to the fMRI undergo safety screening prior to the procedure. These participants will fill out a standard safety form that records medical history relevant to scanner safety (e.g., implanted devices, surgical history, claustrophobia history). If safety criteria are not met, they are not eligible for fMRI and the procedure is not performed.

Table 3 summarizes the participant timeline for the TRE study, including enrollment, intervention, assessments, and adherence and monitoring.

**Table 3:** Participant timeline for the time-restricted eating study protocol.

		STUDY PERIOD														
	Enrollment	Study visit 1			Follow-up											Study visit 2
TIMEPOINT (weeks)	-4 to -1	0		2	4	6	8	10	12	14	16	18	20	22	24	26±2
**ENROLLMENT:**																
***Eligibility screening***	X															
***Informed consent*** *	X	X														
**INTERVENTION:**																
***Time-restricted eating***			<————————————————————————————————————————->													
**ASSESSMENTS:**																
***Intervention orientation***		X														
***Demographics questionnaire***		X														
***Medical history questionnaire***		X														Update
***Physical activity questionnaire***		X														X
***24-h dietary recall***				X	X	X	X	X	X	X	X	X	X	X	X	
***Cognitive battery***		X														X
***Blood draw***		X														X
***Bioimpedance scan***		X														X
***Dual X-ray absorptiometry*** **		X														X
***Functional near-infrared spectroscopy***		X														X
***Electroencephalography***		X														X
***Brain imaging*** **		X														X
***Dynamic retinal vessel analysis***		X														X
***Brachial artery sonography***		X														X
***Laser speckle contrast imaging***		X														X
***Sublingual vasculature assessment***		X														X
***Pulse wave analysis***		X														X
**ADHERENCE AND MONITORING:**																
***Fasting diary***			<————————————————————————————————————————->													
***Check-in & adverse events reporting***				X	X	X	X	X	X	X	X	X	X	X	X	X

* Participants can choose to have separate consent and baseline visits or a single consent plus baseline visit

** These assessments are optional and, if opted in, occur at 0+2 and 26±2 weeks

### Data management and monitoring

All clinical and digital data obtained during the study are de-identified and stored electronically in HIPAA-compliant storage including institutional shared drive, password-protected encrypted external hard drives, and user-authenticated REDCap database. Access to participant data is limited to study staff only. Physical records are kept in a locked cabined located in a secure room that is only accessible to the research team. Further, a safety officer approved by the sponsor and Institutional Review Board monitors the study data and safety according to the approved Data and Safety Monitoring Plan. They receive biannual reports as well as ongoing reports of harms for classification and monitoring.

### Statistical methods

All data will be presented as mean ± standard deviation or standard error, as applicable. Macro- and microvascular endothelial function data will be corrected for mean arterial pressure and analyzed as previously described [32]. For all statistical analyses, we will use visit 1 and visit 2 as repeated measures. We will perform normality tests and then compare baseline and endpoint data using paired t-test or Wilcoxon matched pairs, as appropriate. Regression models will be used to study the association between energy balance and cerebrovascular and endothelial function outcomes with adjusting for confounding factors (Table 3). Missing data will be handled using multiple imputation due to its robustness for small sample sizes. For post-hoc analysis, we will perform the Bonferroni test. The level of significance will be set at less than 5%. fNIRS data will be analyzed using a pipeline based on the general linear model (GLM) approach created using the Brain AnalyzIR toolbox as described previously [24, 37]. EEG data will be analyzed using a pipeline created in the EEGLAB toolbox, as previously described [38]. fNIRS and EEG data will be analyzed in MATLAB (2023b or later version, Mathworks, Natick, MA). Other statistical analyses will be performed in Prism (10 or later version, GraphPad Software, La Jolla, CA).

## Discussion

We expect that adhering to a 10:14 TRE regimen for 6 months will significantly improve cerebrovascular and endothelial function in community-dwelling older adults, and that this will be accompanied by improvements in cognitive performance. These results would indicate that TRE is a potential dietary intervention to improve age-related cerebrovascular dysfunction and cognitive impairment. This would provide evidence support for the application of TRE as a non-pharmacological therapeutic strategy to delay the incidence or slow the progression of cognitive impairment in older adults.

We selected a 10:14 regimen considering that previous studies on the effect of TRE in older adults that utilized a shorter eating window (8 h) reported significant weight loss [15–17, 39], which–besides not being an outcome in this study–may be undesirable in this population. Short eating windows (6–8 h) may also be detrimental for skeletal muscle anabolism and worsen muscle mass loss in older adults [40]. Further, recent data suggest that an 8-h eating window TRE regimen negatively affects social activities among adults [41], and social engagement improves quality of life and several health outcomes in older adults [42]. Thus, we selected a 10-h eating window TRE regimen as the most appropriate for older adults to minimize adverse events.

Although older age is defined as starting at 65 years of age, large population-based studies show that endothelial function start to rapidly decline after 50 years of age (33–35). Further, cognitive decline is a gradual process that starts before the manifestation of clinical signs and symptoms, with about 11% of US adults over 45 years of age experiencing subjective cognitive decline [43]. Therefore, our study will recruit individuals between 55 and 80 years of age and will not include the oldest old adults (>80 years of age) because their inclusion may affect data interpretation (enrollment of survivors) and because safety and tolerability of TRE on this age group is not established.

### Limitations

This study has important limitations that must be acknowledged. First, this is a single-arm study without a control arm; second, the sample size is small; and third, there may be unmeasured confounders that may introduce bias to the analysis. These limitations limit the generalizability of the study and will be thoroughly considered when reporting the final results.

## Supporting information

S1 File Completed SPIRIT checklist. (PDF)

S2 File IRB-approved protocol. (PDF)

## References

[1] Manly JJ, Jones RN, Langa KM, Ryan LH, Levine DA, McCammon R, et al. Estimating the Prevalence of Dementia and Mild Cognitive Impairment in the US: The 2016 Health and Retirement Study Harmonized Cognitive Assessment Protocol Project. JAMA Neurol. 2022;79(12):1242–9. doi: 10.1001/jamaneurol.2022.3543 36279130 PMC9593315

[2] Global Burden of Disease Dementia Collaborators. Global, regional, and national burden of Alzheimer’s disease and other dementias, 1990–2016: a systematic analysis for the Global Burden of Disease Study 2016. Lancet Neurol. 2019;18(1):88–106. doi: 10.1016/S1474-4422(18)30403-4 30497964 PMC6291454

[3] Zissimopoulos JM, Tysinger BC, St Clair PA, Crimmins EM. The Impact of Changes in Population Health and Mortality on Future Prevalence of Alzheimer’s Disease and Other Dementias in the United States. J Gerontol B Psychol Sci Soc Sci. 2018;73(suppl_1):S38–S47. doi: 10.1093/geronb/gbx147 29669100 PMC6019010

[4] Gorelick PB, Scuteri A, Black SE, Decarli C, Greenberg SM, Iadecola C, et al. Vascular contributions to cognitive impairment and dementia: a statement for healthcare professionals from the american heart association/american stroke association. Stroke. 2011;42(9):2672–713. doi: 10.1161/STR.0b013e3182299496 21778438 PMC3778669

[5] Toth P, Tarantini S, Csiszar A, Ungvari Z. Functional vascular contributions to cognitive impairment and dementia: mechanisms and consequences of cerebral autoregulatory dysfunction, endothelial impairment, and neurovascular uncoupling in aging. Am J Physiol Heart Circ Physiol. 2017;312(1):H1–H20. doi: 10.1152/ajpheart.00581.2016 27793855 PMC5283909

[6] Katusic ZS, d’Uscio LV, He T. Emerging Roles of Endothelial Nitric Oxide in Preservation of Cognitive Health. Stroke. 2023;54(3):686–96. doi: 10.1161/STROKEAHA.122.041444 36848426 PMC9991080

[7] Sorond FA, Schnyer DM, Serrador JM, Milberg WP, Lipsitz LA. Cerebral blood flow regulation during cognitive tasks: effects of healthy aging. Cortex. 2008;44(2):179–84. doi: 10.1016/j.cortex.2006.01.003 18387547 PMC2398722

[8] Dichgans M, Leys D. Vascular Cognitive Impairment. Circ Res. 2017;120(3):573–91. doi: 10.1161/CIRCRESAHA.116.308426 28154105

[9] Tarumi T, Zhang R. Cerebral blood flow in normal aging adults: cardiovascular determinants, clinical implications, and aerobic fitness. J Neurochem. 2018;144(5):595–608. doi: 10.1111/jnc.14234 28986925 PMC5874160

[10] Zhu WM, Neuhaus A, Beard DJ, Sutherland BA, DeLuca GC. Neurovascular coupling mechanisms in health and neurovascular uncoupling in Alzheimer’s disease. Brain. 2022;145(7):2276–92. doi: 10.1093/brain/awac174 35551356 PMC9337814

[11] Attwell D, Buchan AM, Charpak S, Lauritzen M, Macvicar BA, Newman EA. Glial and neuronal control of brain blood flow. Nature. 2010;468(7321):232–43. doi: 10.1038/nature09613 21068832 PMC3206737

[12] Claassen JAHR, Thijssen DHJ, Panerai RB, Faraci FM. Regulation of cerebral blood flow in humans: physiology and clinical implications of autoregulation. Physiological Reviews. 2021;101(4):1487–559. doi: 10.1152/physrev.00022.2020 33769101 PMC8576366

[13] Willie CK, Tzeng YC, Fisher JA, Ainslie PN. Integrative regulation of human brain blood flow. J Physiol. 2014;592(5):841–59. doi: 10.1113/jphysiol.2013.268953 24396059 PMC3948549

[14] Kortas JA, Reczkowicz J, Juhas U, Ziemann E, Swiatczak A, Prusik K, et al. Iron status determined changes in health measures induced by nordic walking with time-restricted eating in older adults- a randomised trial. BMC Geriatr. 2024;24(1):300. doi: 10.1186/s12877-024-04876-8 38553690 PMC10979559

[15] Domaszewski P, Konieczny M, Dybek T, Lukaniszyn-Domaszewska K, Anton S, Sadowska-Krepa E, et al. Comparison of the effects of six-week time-restricted eating on weight loss, body composition, and visceral fat in overweight older men and women. Exp Gerontol. 2023;174:112116. doi: 10.1016/j.exger.2023.112116 36739795

[16] Domaszewski P, Konieczny M, Pakosz P, Lukaniszyn-Domaszewska K, Mikulakova W, Sadowska-Krepa E, et al. Effect of a six-week times restricted eating intervention on the body composition in early elderly men with overweight. Sci Rep. 2022;12(1):9816. doi: 10.1038/s41598-022-13904-9 35701451 PMC9198237

[17] Domaszewski P, Konieczny M, Pakosz P, Baczkowicz D, Sadowska-Krepa E. Effect of a Six-Week Intermittent Fasting Intervention Program on the Composition of the Human Body in Women over 60 Years of Age. Int J Environ Res Public Health. 2020;17(11). doi: 10.3390/ijerph17114138 32531956 PMC7312819

[18] Anton SD, Lee SA, Donahoo WT, McLaren C, Manini T, Leeuwenburgh C, et al. The Effects of Time Restricted Feeding on Overweight, Older Adults: A Pilot Study. Nutrients. 2019;11(7). doi: 10.3390/nu11071500 31262054 PMC6682944

[19] Martens CR, Rossman MJ, Mazzo MR, Jankowski LR, Nagy EE, Denman BA, et al. Short-term time-restricted feeding is safe and feasible in non-obese healthy midlife and older adults. Geroscience. 2020;42(2):667–86. doi: 10.1007/s11357-020-00156-6 31975053 PMC7206473

[20] Saini SK, Singh A, Saini M, Gonzalez-Freire M, Leeuwenburgh C, Anton SD. Time-Restricted Eating Regimen Differentially Affects Circulatory miRNA Expression in Older Overweight Adults. Nutrients. 2022;14(9). doi: 10.3390/nu14091843 35565812 PMC9100641

[21] Andriessen C, Fealy CE, Veelen A, van Beek SMM, Roumans KHM, Connell NJ, et al. Three weeks of time-restricted eating improves glucose homeostasis in adults with type 2 diabetes but does not improve insulin sensitivity: a randomised crossover trial. Diabetologia. 2022;65(10):1710–20. doi: 10.1007/s00125-022-05752-z 35871650 PMC9477920

[22] Butcher NJ, Monsour A, Mew EJ, Chan AW, Moher D, Mayo-Wilson E, et al. Guidelines for Reporting Outcomes in Trial Protocols: The SPIRIT-Outcomes 2022 Extension. JAMA. 2022;328(23):2345–56. doi: 10.1001/jama.2022.21243 36512367

[23] Mirmiran P, Bahadoran Z, Gaeini Z. Common Limitations and Challenges of Dietary Clinical Trials for Translation into Clinical Practices. Int J Endocrinol Metab. 2021;19(3):e108170. doi: 10.5812/ijem.108170 34567133 PMC8453651

[24] Mukli P, Pinto CB, Owens CD, Csipo T, Lipecz A, Szarvas Z, et al. Impaired Neurovascular Coupling and Increased Functional Connectivity in the Frontal Cortex Predict Age-Related Cognitive Dysfunction. Advanced Science. 2024;11(10):2303516. doi: 10.1002/advs.202303516 38155460 PMC10962492

[25] Stewart AL, Mills KM, King AC, Haskell WL, Gillis D, Ritter PL. CHAMPS physical activity questionnaire for older adults: outcomes for interventions. Med Sci Sports Exerc. 2001;33(7):1126–41. doi: 10.1097/00005768-200107000-00010 11445760

[26] Subar AF, Kirkpatrick SI, Mittl B, Zimmerman TP, Thompson FE, Bingley C, et al. The Automated Self-Administered 24-hour dietary recall (ASA24): a resource for researchers, clinicians, and educators from the National Cancer Institute. J Acad Nutr Diet. 2012;112(8):1134–7. doi: 10.1016/j.jand.2012.04.016 22704899 PMC3721511

[27] Kirkpatrick SI, Subar AF, Douglass D, Zimmerman TP, Thompson FE, Kahle LL, et al. Performance of the Automated Self-Administered 24-hour Recall relative to a measure of true intakes and to an interviewer-administered 24-h recall. Am J Clin Nutr. 2014;100(1):233–40. doi: 10.3945/ajcn.114.083238 24787491 PMC4144101

[28] Krugh M, Langaker M. Dual-Energy X-Ray Absorptiometry. StatPearls. 2024. Available from: https://www.ncbi.nlm.nih.gov/books/NBK519042/30085584

[29] Parsey CM, Bagger JE, Trittschuh EH, Hanson AJ. Utility of the iPad NIH Toolbox Cognition Battery in a clinical trial of older adults. J Am Geriatr Soc. 2021. doi: 10.1111/jgs.17382 34342879 PMC8648969

[30] Whelton PK, Carey RM, Aronow WS, Casey DE, Jr., Collins KJ, Dennison Himmelfarb C, et al. 2017 ACC/AHA/AAPA/ABC/ACPM/AGS/APhA/ASH/ASPC/NMA/PCNA Guideline for the Prevention, Detection, Evaluation, and Management of High Blood Pressure in Adults: A Report of the American College of Cardiology/American Heart Association Task Force on Clinical Practice Guidelines. Hypertension. 2018;71(6):e13–e115. doi: 10.1161/HYP.0000000000000065 29133356

[31] Harris RA, Nishiyama SK, Wray DW, Richardson RS. Ultrasound assessment of flow-mediated dilation. Hypertension. 2010;55(5):1075–85. doi: 10.1161/HYPERTENSIONAHA.110.150821 20351340 PMC2878744

[32] Csipo T, Lipecz A, Fulop GA, Hand RA, Ngo BTN, Dzialendzik M, et al. Age-related decline in peripheral vascular health predicts cognitive impairment. GeroScience. 2019;41(2):125–36. doi: 10.1007/s11357-019-00063-5 31030329 PMC6544701

[33] Kotliar KE, Lanzl IM, Schmidt-Trucksäss A, Sitnikova D, Ali M, Blume K, et al. Dynamic retinal vessel response to flicker in obesity: A methodological approach. Microvasc Res. 2011;81(1):123–8. doi: 10.1016/j.mvr.2010.11.007 21094174

[34] Garhofer G, Bek T, Boehm AG, Gherghel D, Grunwald J, Jeppesen P, et al. Use of the retinal vessel analyzer in ocular blood flow research. Acta Ophthalmol. 2010;88(7):717–22. doi: 10.1111/j.1755-3768.2009.01587.x 19681764

[35] Lipecz A, Csipo T, Tarantini S, Hand RA, Ngo BN, Conley S, et al. Age-related impairment of neurovascular coupling responses: a dynamic vessel analysis (DVA)-based approach to measure decreased flicker light stimulus-induced retinal arteriolar dilation in healthy older adults. Geroscience. 2019;41(3):341–9. doi: 10.1007/s11357-019-00078-y 31209739 PMC6702523

[36] Kotliar K, Hauser C, Ortner M, Muggenthaler C, Diehl-Schmid J, Angermann S, et al. Altered neurovascular coupling as measured by optical imaging: a biomarker for Alzheimer’s disease. Sci Rep. 2017;7(1):12906. doi: 10.1038/s41598-017-13349-5 29018233 PMC5635105

[37] Santosa H, Zhai XT, Fishburn F, Huppert T. The NIRS Brain AnalyzIR Toolbox. Algorithms. 2018;11(5). doi: 10.3390/a11050073 38957522 PMC11218834

[38] Delorme A, Makeig S. EEGLAB: an open source toolbox for analysis of single-trial EEG dynamics including independent component analysis. J Neurosci Methods. 2004;134(1):9–21. doi: 10.1016/j.jneumeth.2003.10.009 15102499

[39] Zhao L, Hutchison AT, Liu B, Yates CL, Teong XT, Wittert GA, et al. Time-restricted eating improves glycemic control and dampens energy-consuming pathways in human adipose tissue. Nutrition. 2022;96:111583. doi: 10.1016/j.nut.2021.111583 35150947

[40] Lees MJ, Hodson N, Moore DR. A muscle-centric view of time-restricted feeding for older adults. Curr Opin Clin Nutr Metab Care. 2021;24(6):521–7. doi: 10.1097/MCO.0000000000000789 34475325

[41] Antoni R, Robertson T., Robertson M., & Johnston J. A pilot feasibility study exploring the effects of a moderate time-restricted feeding intervention on energy intake, adiposity and metabolic physiology in free-living human subjects. J Nutr Sci. 2018;7(E22). doi: 10.1017/jns.2018.13

[42] Bath PA, Deeg D. Social engagement and health outcomes among older people: introduction to a special section. Eur J Ageing. 2005;2(1):24–30. doi: 10.1007/s10433-005-0019-4 28794713 PMC5547666

[43] Centers for Disease Control and Prevention. Subjective Cognitive Decline—A Public Health Issue. 2019. Available from: https://www.cdc.gov/aging/data/subjective-cognitive-decline-brief.html

