# Supplementary material for: Time-restricted eating for prevention of age-related vascular cognitive decline in older adults: A protocol for a single-arm open-label interventional trial
Source: PLoS One. 2024 Dec 9;19(12):e0314871. doi: 10.1371/journal.pone.0314871 (PMC11627372; doi:10.1371/journal.pone.0314871)
Supplement: S2 File — (PDF) [file pone.0314871.s002.pdf]

PI Name: Andriy Yabluchanskiy, MD, PhD
August 15, 2024

Title of Project: Time restricted eating for prevention of age-related vascular cognitive decline in older adults

Principal Investigator: Andriy Yabluchanskiy, MD, PhD

Abstract

This proposal, which focuses on the effects of a readily translatable and attainable dietary lifestyle intervention for aged individuals (time restricted eating [TRE]) against age-related microvascular endothelial dysfunction, is a rational avenue to investigate the effects of modifiable lifestyle interventions to ameliorate cognition and health-span in the Oklahoma elderly population. Caloric restriction (CR) exerts multifaceted anti-aging and lifespan extending effects and has been demonstrated to be an effective nutritional intervention that can improve vascular health and cognitive function. However, adherence to CR remains a challenge and a translational barrier as most humans may not be able or willing to reduce their caloric intake by 30% over extensive periods of time. Intermittent fasting can recapitulate the benefits of CR without limiting calorie intake in older adults. TRE is considered the best approach to intermittent fasting for elderly individuals as it allows consumption of required calories within a condensed daily eating window (4 to 10 hours), resulting in the greatest fasting stimulus without a net reduction in calorie intake. Guided by our published results and strong preliminary data, we propose to elucidate the role of TRE in aging-induced impairment of NVC responses and dysregulation of CBF, offering new targets for prevention and treatment of age-related vascular cognitive impairment and related dementias. To test our hypothesis, we propose the following specific aims: 1) Determine the impact of TRE on microvascular endothelial function, cognitive function, and NVC responses and microvascular endothelial in healthy adults, and 2) Determine the impact of TRE on circulating biomarkers and cerebromicrovascular endothelial cell SIRT1 activity. Our expectation is that our findings will provide background for novel therapeutic approaches for prevention/treatment of cognitive impairment in high-risk elderly patients.

Human Research Participant Protection
The University of Oklahoma
IRB APPROVED
AAHRPP
IRB NUMBER: 16102
IRB APPROVAL DATE: 09/14/2024

A. Specific Aims

Our central hypothesis is that closer adherence to TRE will improve endothelial function, NVC responses, resulting in improved cognitive performance, potentially through activation of SIRT1-dependent vasoprotective pathways. The following aims are proposed:

Aim 1: **Determine the impact of TRE on microvascular endothelial function, cognitive function, and NVC responses in older adults.** Study 1 will evaluate NVC responses using functional near-infrared spectroscopy (fNIRS), electroencephalography (EEG), and dynamic retinal vessel analysis (DVA). Study 2 will evaluate peripheral micro- and macrovascular endothelial function using laser speckle contrast imaging and flow-mediated dilation approaches. Study 3 will evaluate performance in different cognitive domains using a comprehensive battery of cognitive tests.

Aim 2: **Determine the impact of TRE on circulating biomarkers and cerebromicrovascular endothelial cell SIRT1 activity.** Irrespective of whether the results of Aim 1 are positive or negative, we need to understand the effect of TRE on SIRT1 activity levels and related deacetylation by-products in the human participants. Study 4 To assess the effects of TRE on cellular SIRT1 activity we will treat human cerebral microvascular endothelial cells (CMVECs) with fasting serum obtained from older adults undergoing TRE and age-matched controls. We anticipate that altered SIRT1 activation will affect NO bioavailability, endothelial oxidative stress, and inflammation in CMVECs and in circulating blood. We also hypothesize that these changes will correlate the magnitude of NVC impairment and cognitive performance in older adults, and that TRF treatment l will improve these outcomes.

B. Background and Significance

As life expectancy rises, VCID has become a leading public health issue in the developed countries. VCID is the second most common cause of dementia after Alzheimer's disease (AD), and accounts for close to 50% of cognitive impairment cases in the elderly [1]. Additionally, vascular contributions to cognitive impairment and dementia are now considered critical for AD as well. There is strong clinical evidence that impaired blood supply to the brain plays a critical role in cognitive decline in the elderly [2-7]. The clinical findings of aging-induced dysregulation of CBF and cognitive impairment have also been confirmed in animal models (Fig 2) [8-12]. To date, no interventional strategies exist for the prevention or treatment of aging induced CBF dysregulation and cognitive decline. Because the energetic demands of neurons are high and the brain has little reserve capacity, maintenance of normal brain function is critically dependent on moment-to-moment adjustment of CBF [13-16]. This requirement is fulfilled by NVC, a vital mechanism that maintains an optimal cerebral tissue microenvironment, enabling rapid increases in O_2 and glucose delivery and washout of toxic metabolites during periods of intense neuronal activity. Aging results in significant impairment of cerebromicrovascular endothelial function, which promotes NVC dysfunction which are causally linked to the genesis of cognitive decline [13, 17, 18]. It is believed that TRE mimics several aspects of the beneficial biological effects of calorie restriction [19]. Importantly, TRE can improve endothelial function. Our contribution is expected to be a demonstration that TRE confers endothelial rejuvenating effects in the cerebral microcirculation by inducing SIRT1-dependent pathways, which results in restoration of NVC responses and increased CBF. TRE-mediated cerebromicrovascular geroprotection is expected to improve cognitive function in aging.

C. Preliminary Studies/Progress Report

Currently, there is not pilot results from human participants.

D. Research Design and Methods

Clinical trial design: We will test the primary hypothesis that TRE improves NVC responses and endothelial function in older adults. This hypothesis will be tested by assessing the effects of TRE (10 hours eating window) in community dwelling older adults (55-80 years of age) in a 6-month study. At screening (Visit 0), participants will be evaluated for eligibility criteria, after obtaining informed consent. Individuals

Human Research Participant Protection The University of Oklahoma IRB APPROVED AAHRPP
IRB NUMBER: 16102
IRB APPROVAL DATE: 09/14/2024

will be asked report medications or supplements intake. Screening assessments and baseline physiological assessments may be conducted at that same visit (baseline, Visit 1) if all eligibility criteria are met, or within 4 weeks of Visit 0 (screening window) at the discretion of the investigator, when eligibility will be again confirmed. After baseline, the first fMRI and dual X-ray absorptiometry (DXA) scan visits will be performed, if the participant opts in to these measurements.

Upon completion of baseline physiologic measurements, all participants will be instructed to maintain TRE for the ensuing 6 months. Participants will be instructed on how to adhere to the TRE protocol. Participants will be evaluated at the Translational Geroscience Laboratory at the end of the protocol, at month 6 (-14/+14 days), when all physiologic assessment will be repeated (Visit 2). If participants terminate the protocol early for any reason, they will be invited to an early termination visit (ETV), which will include repeat physiologic and other evaluations same as in Visit 2. After visit 2, the second fMRI and DXA scan visits will be performed. As stated in the protocol and consent form, MRI and DXA scan visits are optional.

***Power analysis**:* Based on our preliminary data analysis, a total of 27 eligible participants will provide 80% power for detecting a decrease of 7.4 in fNIRS against the null hypothesis of no change. A standard deviation of 13.1 was used in the calculation. After adjusting for a 20% dropout, the total of 32 participants will be enrolled. We will use intent-to-treat approach, wherein every participant will be included in the analysis regardless of whether participant is on or off the TRE for the final visit. To control for potential confounding, we will account for BMI, depression, the use of opiates, and other non-exclusive chronic conditions.

Adherence protocols: Adherence to study protocol will be monitored using daily surveys of eating periods. All participants will be provided with a fasting diary (electronically or using the sample diary included in supplementary information) in which they will the time that they started and stopped eating each day.

Dietary intake and physical activity assessments: Dietary intake data for 24-hour recalls will be collected and analyzed using the Automated Self-Administered 24-hour (ASA24) Dietary Assessment Tool, version 2022, developed by the National Cancer Institute (https://epi.grants.cancer.gov/asa24) or using interviewer-led 5-pass USDA method of dietary recall via phone or in-person during visits. The ASA24 system does not capture any personally identifiable data from respondents and respondents' data are protected by industry standard security protocols. The ASA24 tool is validated for use in the general US population and has comparable performance to interviewer-administered 24-h recalls [20]. Participants will complete one 24-h recall biweekly for a total of 12 recalls. Recalls will be scheduled at random to cover different days of the week. Recall data will be used to estimate participants' dietary intake during the intervention. Physical activity level (PAL) will be assessed and classified into sedentary, lightly active, active, and very active using the self-administered CHAMPS Physical Activity Questionnaire for Older Adults. This tool is validated for use in older US adults [21].

Body composition and physical exam: Weight and body composition will be acquired using a bioimpedance scale (Tanita Body Composition Analyzer BC-418) at each study visit. Total daily energy expenditure (TDEE) will be estimated using the Mifflin-St. Jeor equation [22-24] multiplied by the appropriate factor according to PAL. Estimated dietary intakes and TDEE will be used to determine whether a participant was in a hypocaloric, eucaloric, or hypercaloric state during the intervention. Moreover, we will collect data on blood pressure and heart rate. Blood glucose and cholesterol will be determined using the Lipid Panel Plus panel from Piccolo Xpress. Optionally, participants may choose to undergo a DXA scan to obtain body composition (% fat mass and % lean mass) and bone density estimates. This medical imaging technique uses low levels of X-rays to measure bone density and body composition. DXA scans will take place at the Oklahoma Shared Clinical and Translational Resources (OKC, OK).

Human Research Participant Protection The University of Oklahoma IRB APPROVED AAHRPP
IRB NUMBER: 16102
IRB APPROVAL DATE: 09/14/2024

Study 1: Determine NVC responses using functional near-infrared spectroscopy (fNIRS), electroencephalography (EEG), and dynamic retinal vessel analysis (DVA)*:*

We will use **fNIRS** recordings to assess NVC responses elicited during the performance of a standardized cognitive n-back test [25]. For the *n-back* task, the participant is required to monitor a series of letters (or numbers) on the computer screen and to respond by clicking the computer mouse right button when the presented letter matches the letter *n*-steps back. We will also record the NVC responses using the fNIRS during standardized motor task, or other standardized stimulation paradigm. We will assess neuronal activity during a similar n-back paradigm (series of numbers) to evaluate the neuronal component of the NVC using the **EEG**.

To directly assess the effect of TRE on NVC responses at the single-vessel level, changes in the diameter of retinal arterioles in response to flickering light stimulation (**DVA**) will be measured, as reported [26].Static vessel analysis will also be performed right after DVA measures. Functional alterations in retinal arterioles closely correlate with those in cerebral microvessels and predict cognitive impairment [26]. All study participants will be evaluated for visual acuity, using a standard Snellen chart (or similar), and for intraocular blood pressure, using a calibrated Goldmann applanation tonometer or the i-Care device (iCare Ic100 – cleared by FDA to be used in human subjects).

The applanation tonometry procedure involves the use of tetracaine hydrochloride eye drop (or similar) to anesthetize the eye, of instilling fluorescein dye in the conjunctival sac and placing a sterilized prism against the cornea. The tonometer tip will be sterilized before each measurement.

Study 2: Determine peripheral micro- and macrovascular endothelial function in study participants before and after TRE*:*

Changes in microvascular blood flow will be measured in the nailbeds of the right hand in response to a 5-minute blood flow occlusion in the brachial artery using a sphygmomanometer cuff inflated to the level of current systolic blood pressure plus 50 mmHg, or a maximum of 200 mmHg using the **laser speckle contrast imaging** (Perimed). The magnitude of the response is a reliable indicator of endothelium-mediated microvascular dilation.

We will also assess macrovascular endothelial function using the gold-standard method of flow-mediated dilation (FMD) in the brachial artery using an 8-12 MHz transducer (Phillips Affinity 70, or other). Reactive hyperemia will be induced by 5 minutes of forearm occlusion with a sphygmomanometer cuff inflated to the level of current systolic blood pressure plus 50 mmHg, or a maximum of 200 mmHg. After the cuff is deflated, changes in the diameter of the brachial artery will be recorded at 20-, 40-, 60-, and 80-seconds, and FMD will be calculated as maximal relative diameter gain normalized to baseline [27].

We will also assess **arterial stiffness** using the pulse wave analysis approach (SphygmoCor, Atcor medical, Itasca IL, or similar). We will also perform a short (approximately 5 minutes long) recording of the sublingual vasculature using the **GlycoCheck** device (Microvascular Health Solutions) – the system is equipped with a high-resolution camera that is capable to evaluate capillary density, blood volume, blood flow and red cell velocity, endothelial glycocalyx function.

Study 3: Evaluate performance in different cognitive domains using a comprehensive battery of cognitive tests. To assess different cognitive domains, a selection of tests from the NIH toolbox will be used as previously described [28, 29]. These batteries of tests are optimized to detect age-related changes in cognition and assesses reaction time, practice (sensorimotor function and comprehension), attention (processing and psychomotor speed, sustained attention), memory (visual episodic and verbal recognition memory, attention, short-term memory, visual recognition), and executive function (working memory and strategy).

Study 4: Blood draw (venipuncture). We will perform a blood draw via venipuncture and collect up to 40mL of blood for each visit.

Our prediction, based on our preclinical preliminary data and other laboratory's findings, is that serum isolated from study participants that have best adhered to a 6-month long TRE regimen will contain several circulating factors that will induce SIRT1 activation in commercially available human CMVECs. This study will simulate *in vitro* the microenvironment and the circulating factors in which cerebral

Human Research Participant Protection The University of Oklahoma IRB APPROVED AAHRPP
IRB NUMBER: 16102
IRB APPROVAL DATE: 09/14/2024

endothelial cells are exposed to *in vivo* within the study participants. To measure SIRT1 in CMVECs, we will perform a nuclear extraction in the resulting endothelial cells followed by immunoprecipitation of SIRT1. SIRT1 activity in the samples will be measured using the Cyclex SIRT1 Deacetylase Fluorimetric Assay as described [30]. Specific activity of SIRT1 will be assessed by measuring time-dependent changes in fluorescence intensity, normalized to protein concentration determined by the Bradford method on the nuclear extracts. Deacetylase activity of SIRT1 will be measured using the PNC1–OPT assay, which provides a quick and reliable method to measure SIRT1 deacetylation activity without specialized equipment [31]. Alternatively, measurements of deacetylation of Sirt1 substrates such as p53 and histone H4 can be performed as described [32].

NO production by CMVECs in response to exogenously administered ATP will be assessed by flow cytometry. Cellular production of reactive oxygen species (ROS) will be measured in detector CMVECs by flow cytometry using the redox sensitive fluorescent dye DHE [15].

In addition, a separate set of plasma, serum samples, whole blood, and blood cells (including white blood cells) will be stored for future analyses.

Once stored in tissue repository bank, blood samples may be used for future studies, including genetic testing.

Study 5: Examine the mechanisms underlying TRE using functional magnetic resonance imaging (fMRI). We will also use fMRI to assess brain changes, including the quantification of cortical reorganization. Using fMRI, we will determine the changes in Blood-Oxygen-Level dependent activation. We will compare these neurophysiological parameters before and after treatment. Participants will perform 2 fMRI visits at the Laureate Institute for Brain Research (Tulsa, OK). fMRI recordings will be performed during a standardized cognitive, sensory and/or motor task.

Future studies: Identifiers might be removed, and the de-identified information may be used for future research without additional informed consent from the subject.

E. Chart Review

N/A.

F. Biospecimens

As part of our study, we will collect blood samples, including whole blood, plasma, serum, and blood cells (e.g. white blood cells). Biospecimens will be collected prospectively, at the time of 1st and 2nd visit. All subjects will be assigned study ID, and all biospecimens will be labeled with corresponding study ID.

Biospecimens will not be used for commercial profit. While it is not planned at the moment, the research may include genetic analysis, such as transcriptomics and single-cell sequencing analysis to determine the effect of TRE on cellular function.

G. Banking/Repository/Database

All biospecimens will be stored in the Translational Geroscience Laboratory in -20 or -80 freezers, specifically purchased to store human biospecimens. All biospecimens will be stored until used. All biospecimens will have study participant ID label and only study personnel will have access to the identifiable data.

Biospecimens may be shared with researchers upon request and upon availability without providing identifiable information.

All electronic data associated with this study, including linkable data to the biospecimens and potential future electronic data from biospecimen analyses will be stored on encrypted drives (OUHSC shared drive and encrypted external hard drives).

H. Inclusion / Exclusion Criteria

Human Research Participant Protection
The University of Oklahoma
IRB APPROVED
AAHRPP
IRB NUMBER: 16102
IRB APPROVAL DATE: 09/14/2024

Table 1: Inclusion criteria:	Exclusion criteria:
• Age ≥55 and ≤80 years of age<br>• Adequate hearing and visual acuity to participate in the examinations<br>• Ability to read and write in English<br>• Competence to provide informed consent<br>• Mini-Montreal Cognitive Assessment score ≥12<br>• Mini Nutrition Assessment score ≥12	• Vision or hearing impairment that would impair the ability to complete study assessments<br>• Active CNS disease including multiple sclerosis, uncontrolled seizures, active brain cancer<br>• Cerebrovascular accident other than TIA within 60 days prior to Visit 0<br>• Major psychiatric disease, including major depression not currently controlled on medications, and alcohol or drug abuse<br>• Diabetics prescribed sulfonylureas, meglitinides, and insulin<br>• Any other medical condition which, in the opinion of investigator, would render the patient inappropriate or too unstable to complete the study protocol

* If the subject is not eligible for an fMRI or does not want to participate, we will not perform this assessment as fMRI is not required for study eligibility and it is only used as a secondary outcome. Similarly, if a participant does not want to participate in a DXA scan, we will not perform this assessment as it is not required for study eligibility.

I. Gender/Minority/Pediatric Inclusion for Research

Both male and female participants will be enrolled in each group, and analysis will account for sex as a biological variable.

J. Recruitment and Enrollment

Patient recruitment will be performed via advertisement through the Oklahoma Clinical and Translational Sciences Institute, via an advertisement in community newspapers and journals that target older adults such as “Senior Living” or other, through the pool of study participants from an ongoing clinical study on aging led by Dr. Yabluchanskiy, through collaborative ties with the Section of Geriatric Medicine, through social media such as Facebook ads, or through other means. We will also use MyChart messaging to advertise the study to potential subjects to increase participant diversity (MyChart message write-up attached to application). Potential subjects may be identified via EPIC’s Best Practice Advisory (BPA) system. While the patient is being seen for a standard of care visit, a notification may be sent to the provider via EPIC’s BPA indicating that the patient may qualify for the research study. This notification would contain a small explanation of the study, why the patient qualifies, and ask the provider to mention it to the patient and indicate the patient’s interest/disinterest. Should the provider mark the patient is “interested”, the study team will be automatically notified and reach out to the patient for initial contact. Electronic health records may also be screened to identify potential study participants who have previously agreed to be contacted for future research. These identified patients would then be contacted by key study personnel via telephone utilizing the approved phone script.

Interested subjects will speak directly to study personnel at the site of physician’s office and then contact us to schedule an outpatient visit in the Translational Geroscience Laboratory at the University of Oklahoma Health Sciences Center. The protocol in details addressing all the questions that might appear during the first contact or at the first visit at the Translational Geroscience Laboratory. All subjects must demonstrate understanding of the study prior to beginning study procedures, and will be given as long as they need to decide whether to participate. All subjects may withdraw their consent at any time, for any reason.

All subjects will be given as long as they need to decide whether to participate. All subjects will also be asked to answer the following questions prior to signing the consent:

1) Please describe the study procedures.
2) Why are you doing this study?
3) What will happen if you decide not to do this study?

Human Research Participant Protection
The University of Oklahoma
IRB APPROVED
AAHRPP
IRB NUMBER: 16102
IRB APPROVAL DATE: 09/14/2024

By answering these questions, the subject will be able to demonstrate that they understand the study procedures and that their participation is completely voluntary.

K. Risks and Benefits

Risks associated with assessment methodologies.

This study carries no more than minimal risk that is otherwise associated with routine medical care and testing.

There is a small but minimal risk of loss of confidentiality. All efforts will be made to protect participant confidentiality as outlined below.

There is a small chance that these results could cause psychological stress for these individuals, and study staff will try to mitigate this risk by demonstrating empathy and support with referral to an appropriate healthcare provider.

The two neurovascular coupling studies utilized in this study are noninvasive. There is a small chance of mild physical discomfort with fNIRS and DVA, and participants may elect to discontinue these procedures if they experience discomfort.

As previously described in IRB protocol #9384: "Flickering light used for the dynamic vessel analysis might trigger a seizure if the participant has photosensitive epilepsy. Individuals with history of seizures will be excluded from this assessment. In addition, dilation of the pupil may lead to an acute angle-closure glaucoma attack. Only participants with a non-occludable angle and with no optic neuropathy (based on the slit lamp examination, these procedures do not require pupil dilation) will receive the topical tropicamide. The participants will be asked to seek immediate medical attention if they experience symptoms of glaucoma attack (severe ocular pain and redness, decreased vision, colored halos in combination with headache, nausea and vomiting)."

Participants will be asked to have a designated driver assist them with transportation home after the study and will be advised that they may have visual difficulty limiting their ability to work for several hours after completion of the dilation.

There are no known harmful side effects associated with temporary exposure to the strong magnetic field used by MRI scanners. However, there are important safety concerns to consider before performing or undergoing an MRI scan. For that reason, a standard Safety Form will be filled out by the radiation technician that records the subject's medical history relevant to scanner safety. Additional sources of risk include the following:

- The subject may experience discomfort being in the confined environment of the scanner.
- The strong magnetic field will affect electronic, magnetic, and metal devices that subjects carry with them or that have been implanted in the subject's body. These will be assessed prior to scanning, and the session will be cancelled if safety criteria are not satisfied.
- Claustrophobia: Some subjects may get anxious in confined spaces.
- Peripheral Nerve Stimulation effects: Occasionally, some subjects may experience muscle twitching or paresthesias, especially in the torso.
- Dizziness: Some individuals may experience light-headedness while in the scanner or when rising from the MRI gurney too rapidly.
- Hearing: MRI scanning produces a loud high frequency tone that can cause hearing damage if appropriate hearing protection is not used. Regarding acoustic noise associated with MRI, all the subjects scanned will receive ear protection (earplugs and/or headphones).

If the subject is not eligible for an MRI at the time of the study, we will not perform this assessment as MRI is not required for study eligibility and it is only used as a secondary outcome.

DXA scans use a minimal amount of radiation (lower than a standard X-ray) and pose no more than minimal risk.

Benefits of the study

Human Research Participant Protection
The University of Oklahoma
IRB APPROVED
AAHRPP
IRB NUMBER: 16102
IRB APPROVAL DATE: 09/14/2024

Participants may derive personal satisfaction by contributing to the overall knowledge on the mechanisms involved in neurodegeneration. They may also receive directly benefits by learning more about their general health, cognitive function and neurovascular function in response to cognitive tasking. If during a study visit, an incidental medical problem is newly uncovered, the PI team will contact the participant's physician after obtaining permission from the subject. Incidental findings from the optional MRI scan will be reviewed by the study team neurologist (Faddi Saleh Velez, MD). Findings from this study will also provide important knowledge to caregivers that may result in modifications to the implemented care and improve clinical outcomes.

L. Multiple Sites

N/A

M. Statistical Methods

There are currently no published data available on the effect of TRE on NVC responses in healthy young and older adults. Data obtained from this project will be used as preliminary data for an NIH application.

Functional near infrared spectroscopy and dynamic retinal vessel analysis: fNIRS data will be analyzed using a pipeline based on General Linear Model (GLM) approach created using the Brain AnalyzIR toolbox. After conversion of optical densities to change of hemoglobin concentration using the Beer-Lambert law, pre-whitening of data with an autoregressive model-based algorithm, and a discrete cosine transformation using high-pass filter, design matrices will be convolved with a canonical hemodynamic response function to predict brain activation. Parameter estimates (beta-weights), scaling the predictors, will be then used for group level statistics. Group level statistics will be performed using a repeated measures mixed effects model. The model will be defined in a Wilkinson-Rogers formula of 'beta ~ -1 + Group +(1|Subject)'. Output of the mixed effects model statistics will be used for a t test. Increased activation/NVC responses will be considered significant where false discovery rate corrected $q<0.05$. When appropriate, beta values from corresponding channels to the cognitive task will be summarized using mean (±SEM) by group and study visit. We will use repeated measures mixed effects models (with a random subject effect) to assess the main effect of group (TRE vs. control) on NVC responses.

For DVA analysis, each participant will be evaluated for mean maximal arteriolar dilation and mean maximal venular dilation in response to flicker light stimulation. The statistical mean of 3 consecutive examinations will be calculated for each subject and each evaluated parameter as previously described. Data will be summarized using mean (±SEM) by group and study visit. We will use GLM with control and TRE treatment as between-group factor, and Visit 1 and Visit 2 as repeated measures (within group) factor. For post-hoc analysis, we will perform the Bonferroni test. The level of significance will be regarded as $p<0.05$.

For EEG analysis, the power spectral density of EEG records (obtain during n-back stimulation) will be estimated using the Welch's method and then averaged across 14 channels of the Emotive EPOC research instrument. Spectra will be plotted in a semilogarithmic graph as a function of EEG frequency. For channel-wise comparison, Lilliefors tests will be applied to verify normality of the data. For normally distributed data, F test will be then used to confirm equality of variances between groups, and the two-sample t test or the Welch-corrected t test will be applied for equal or unequal variances, respectively. For not normally distributed data, a Mann–Whitney U test will be used for group comparison. The false discovery rate (FDR) method of Benjamini and Hochberga will be applied to control for multiple comparisons.

Peripheral endothelial function: For analysis of micro- and macrovascular endothelial function, all data will be corrected for mean arterial pressure. Laser speckle data will be normalized to an average of 3 measurements of skin temperature taken before the start of the assessment. For microvascular endothelial function, we will evaluate the maximal perfusion over the baseline perfusion (x-fold change) and the acute

Human Research Participant Protection
The University of Oklahoma
IRB APPROVED
AAHRPP
IRB NUMBER: 16102
IRB APPROVAL DATE: 09/14/2024

reperfusion index (over the first 4 seconds of cuff release) in the nailbeds and the hand as previously described. For macrovascular endothelial function, change in the brachial artery diameter over the baseline will be calculated (% change), as previously described. When appropriate, data will be summarized using mean (±SEM) by group and study visit. We will use GLM for between-group factor and repeated measures (within group) factor. For post-hoc analysis, we will perform the Bonferroni test. The level of significance will be regarded as $p<0.05$.

Cognitive performance: For cognitive assessment, all NIH Toolbox tests will be organized into two groups of fluid abilities (i.e., working memory, processing speed, episodic memory, and two aspects of executive functioning) and crystallized abilities (i.e., dependent upon past learning and experience), resulting in Standard Scores for these superordinate categories, as well as a total Composite score of all tests. We will use Standard Scores and Composite scores for analysis. These norms were previously reported to align with the age-corrected normative data for the traditional neuropsychological measures, and also because the time interval between baseline Visit 1 and final Visit 2 would not be affected by the participant's race/ethnicity or educational level. We will use GLM for between-group factor and repeated measures (within group) factor. For post-hoc analysis, we will perform the Bonferroni test. The level of significance will be regarded as $p<0.05$.

Functional magnetic resonance imaging (fMRI) will be used to quantify patterns of activation associated with brain networks reorganization before and after treatment on each participant. Structural and functional imaging data will be acquired. Structural T1-weighted scans, T2 scans, and scans assessing tissue characterization will be acquired. Functional scans will be acquired with a single-shot EPI sequence. Functional runs will be collected using a cognitive performance task such as the N-back task, and will have a duration of 21 minutes or less. Each MRI session will be less than 2 hours within the magnet. A repeated measures design (baseline and post-TRE) will be conducted for each participant. Additional baseline data will be obtained for secondary correlations. Besides that, a sensitivity analysis testing the comparison of post-TRE only will be additionally conducted.

N. Data and Safety Monitoring Plan

The PI team will provide direct oversight to ensure that this low-risk trial is conducted according to the protocol.

Clinicaltrials.gov

This study is registered on clinicaltrials.gov under identifier NCT06019195.

O. Data Sharing

The primary objective is to publish any significant research results that will be generated during this project in a peer-reviewed journals. The results of this project will be submitted to local, national, and international scientific conferences and meetings and accepted abstracts will be presented either orally or by poster at these meetings. The results will also be disseminated by presentation at seminars organized by the Oklahoma Clinical and Translational Sciences Institute.

The results may also be uploaded to scientific online repositories such as PhysioNet as requested by the journal. In that case, all data will be de-identified and labeled by group+number (for example Control1, Control2, TRE1, TRE2, these IDs will be different from study IDs stored on the servers at the OUHSC) and supported by subjects' age and sex, treatments/comorbidities.

P. Confidentiality

All clinical and digital data obtained during the study will be stored electronically in a HIPAA compliant storage facility including OUHSC shared drive, encrypted external hard drives, password-protected OUHSC REDCap database. Access to PHI data will be limited to study staff only.

Human Research Participant Protection The University of Oklahoma IRB APPROVED AAHRPP
IRB NUMBER: 16102
IRB APPROVAL DATE: 09/14/2024

Q. Literature Cited

1. Corriveau, R.A., et al., *The Science of Vascular Contributions to Cognitive Impairment and Dementia (VCID): A Framework for Advancing Research Priorities in the Cerebrovascular Biology of Cognitive Decline.* Cell Mol Neurobiol, 2016. **36**(2): p. 281-8.
2. Sorond, F.A., et al., *Neurovascular coupling, cerebral white matter integrity, and response to cocoa in older people.* Neurology, 2013.
3. Sorond, F.A., et al., *Neurovascular coupling is impaired in slow walkers: the MOBILIZE Boston Study.* Ann Neurol, 2011. **70**(2): p. 213-20.
4. Sorond, F.A., et al., *Cerebral blood flow regulation during cognitive tasks: effects of healthy aging.* Cortex, 2008. **44**(2): p. 179-84.
5. Dichgans, M. and D. Leys, *Vascular Cognitive Impairment.* Circ Res, 2017. **120**(3): p. 573-591.
6. Gorelick, P.B. and J.V. Bowler, *Advances in vascular cognitive impairment.* Stroke, 2010. **41**(2): p. e93-8.
7. O'Brien, J.T., et al., *Vascular cognitive impairment.* Lancet Neurol, 2003. **2**(2): p. 89-98.
8. Kisler, K., et al., *Cerebral blood flow regulation and neurovascular dysfunction in Alzheimer disease.* Nat Rev Neurosci, 2017. **18**(7): p. 419-434.
9. Nelson, A.R., et al., *Neurovascular dysfunction and neurodegeneration in dementia and Alzheimer's disease.* Biochim Biophys Acta, 2016. **1862**(5): p. 887-900.
10. Sweeney, M.D., et al., *The role of brain vasculature in neurodegenerative disorders.* Nat Neurosci, 2018. **21**(10): p. 1318-1331.
11. Zlokovic, B.V., *Neurodegeneration and the neurovascular unit.* Nat Med, 2010. **16**(12): p. 1370-1.
12. Zlokovic, B.V., *Neurovascular pathways to neurodegeneration in Alzheimer's disease and other disorders.* Nat Rev Neurosci, 2011. **12**(12): p. 723-38.
13. Tarantini, S., et al., *Pharmacologically-induced neurovascular uncoupling is associated with cognitive impairment in mice.* J Cereb Blood Flow Metab, 2015. **35**(11): p. 1871-81.
14. Toth, P., et al., *Purinergic glio-endothelial coupling during neuronal activity: role of P2Y1 receptors and eNOS in functional hyperemia in the mouse somatosensory cortex.* Am J Physiol Heart Circ Physiol, 2015. **309**(11): p. H1837-45.
15. Toth, P., et al., *Resveratrol treatment rescues neurovascular coupling in aged mice:role of improved cerebromicrovascular endothelial function and down-regulation of NADPH oxidas.* Am J Physiol Heart Circ Physiol, 2014. **306**(3): p. H299-308.
16. Tucsek, Z., et al., *Aging exacerbates obesity-induced cerebromicrovascular rarefaction, neurovascular uncoupling, and cognitive decline in mice.* J Gerontol A Biol Sci Med Sci, 2014. **69**(11): p. 1339-52.
17. Csipo, T., et al., *Assessment of age-related decline of neurovascular coupling responses by functional near-infrared spectroscopy (fNIRS) in humans.* GeroScience, 2019.
18. Sorond, F.A., et al., *Neurovascular coupling, cerebral white matter integrity, and response to cocoa in older people.* Neurology, 2013. **81**(10): p. 904-9.
19. Di Francesco, A., et al., *A time to fast.* Science, 2018. **362**(6416): p. 770-775.
20. Subar, A.F., et al., *The Automated Self-Administered 24-hour dietary recall (ASA24): a resource for researchers, clinicians, and educators from the National Cancer Institute.* J Acad Nutr Diet, 2012. **112**(8): p. 1134-7.
21. Stewart, A. et al., *CHAMPS Physical Activity Questionnaire for Older Adults: Outcomes for Interventions*. Med Sci Sports Exerc, 2001, **33**(7), p. 1126-41.
22. Mifflin, M.D., et al., *A new predictive equation for resting energy expenditure in healthy individuals.* Am J Clin Nutr, 1990. **51**(2): p. 241-7.
23. Frankenfield, D., L. Roth-Yousey, and C. Compher, *Comparison of predictive equations for resting metabolic rate in healthy nonobese and obese adults: a systematic review.* J Am Diet Assoc, 2005. **105**(5): p. 775-89.
24. Porter, J., et al., *Total energy expenditure measured using doubly labeled water compared with estimated energy requirements in older adults (>/=65 y): analysis of primary data.* Am J Clin Nutr, 2019. **110**(6): p. 1353-1361.
25. Sorond, F., Kiely, DK, Galica, A, Moscufo, N, Serrador, JM, Iloputaife, I, Egorova, S, Dell'Oglio, E, Meier, D, Newton, E, Milberg, WP, Guttmann, C, Lipsitz, LA., *Neurovascular Coupling is Impaired in Slow Walkers: The MOBILIZE Boston Study.* Annals of Neurology, 2011. **Revision under review**.
26. Lipecz, A., et al., *Age-related impairment of neurovascular coupling responses: a dynamic vessel analysis (DVA)-based approach to measure decreased flicker light stimulus-induced retinal arteriolar dilation in healthy older adults.* Geroscience, 2019. **41**(3): p. 341-349.
27. Heinen, Y., et al., *Local association between endothelial dysfunction and intimal hyperplasia: relevance in peripheral artery disease.* J Am Heart Assoc, 2015. **4**(2).
28. Csipo, T., et al., *Age-related decline in peripheral vascular health predicts cognitive impairment.* Geroscience, 2019. **41**(2): p. 125-136.
29. Parsey, C.M., et al., *Utility of the iPad NIH Toolbox Cognition Battery in a clinical trial of older adults.* J Am Geriatr Soc, 2021.
30. Csiszar, A., et al., *Anti-oxidative and anti-inflammatory vasoprotective effects of caloric restriction in aging: role of circulating factors and SIRT1.* Mech Ageing Dev, 2009. **130**(8): p. 518-27.
31. Dai, H., et al., *Synthesis and Assay of SIRT1-Activating Compounds.* Methods Enzymol, 2016. **574**: p. 213-244.
32. Minor, R.K., et al., *SRT1720 improves survival and healthspan of obese mice.* Sci Rep, 2011. **1**: p. 70.

Human Research Participant Protection
The University of Oklahoma
IRB APPROVED
AAHRPP
IRB NUMBER: 16102
IRB APPROVAL DATE: 09/14/2024
